# Proactive control for conflict resolution is intact in subclinical obsessive-compulsive individuals

**DOI:** 10.3389/fpsyg.2024.1490147

**Published:** 2024-10-22

**Authors:** Silvia Fornaro, Antonino Visalli, Giada Viviani, Ettore Ambrosini, Antonino Vallesi

**Affiliations:** ^1^Padua Neuroscience Center, University of Padua, Padua, Italy; ^2^Department of Neuroscience, University of Padua, Padua, Italy; ^3^Department of General Psychology, University of Padua, Padua, Italy; ^4^Department of Developmental Psychology and Socialization, University of Padua, Padua, Italy

**Keywords:** obsessive-compulsive traits, proactive control, transdiagnostic, risk factors, conflict resolution, Stroop

## Abstract

**Background:**

Obsessive-compulsive (OC) traits (i.e., tendency to implement stereotyped behaviors to avoid negative consequences) are transversally observed in psychiatric disorders largely differing in terms of clinical manifestations and etiopathogenesis. Interestingly, OC traits were also extensively found in the prodromal phases of the full-blown psychopathology and in healthy relatives of affected individuals. Moreover, OC traits were found to be associated—and possibly underlain by—cognitive control impairments. Nonetheless, the role of such interplay in the onset of OC disorders is yet to be understood. We hypothesized that OC traits are associated with abnormalities in proactively implement cognitive control for solving conflict.

**Methods:**

We administered healthy individuals (*n* = 104) with the perifoveal spatial Stroop task to measure their ability of solving conflict in a proactive fashion, and with Obsessive-Compulsive Inventory (OCI) to stratify population according to the severity of OC traits.

**Results:**

Analysis of response times by means of Linear Mixed-effect models revealed that proactive control performance was not associated with and the severity of OC traits. Furthermore, an equivalence test (Two One-Sided Test) revealed that the association between OCI scores and task performance was equivalent to zero.

**Conclusion:**

These results suggest that the interplay between OC traits and proactive control abnormalities might not contribute to the development of OC-related disorders. Therefore, the role of other cognitive endophenotypes should be scrutinized for exploiting alternative prevention and intervention strategies.

## Introduction

Obsessive-compulsive (OC) traits are broadly described as the tendency to implement stereotyped and repetitive behaviors and thoughts lacking any adaptive functions to avoid perceived negative consequences, accompanied by a subjective feeling of urgency (Figee et al., 2013; Fineberg et al., 2013; Luigjes et al., 2019). Notably, obsessive-compulsive (OC) symptoms and their cognitive and behavioral correlates are trans-diagnostically observed in several mental disorders, fitting in clinical pictures largely differing in terms of their nature, clinical manifestations and underlying causes (Fineberg et al., 2016; Yilmaz et al., 2020; Mandelli et al., 2020; Monaco et al., 2020; Peterson et al., 2022; Zadegan et al., 2023). Interestingly, subclinical OC traits are also extensively present in the healthy population with a non-negligible prevalence and incidence (Fullana et al., 2010; Chamberlain et al., 2018; Tiego et al., 2019). Thus, they negatively impact affected populations—either clinical or subclinical—in terms of quality of life and social functioning, entailing huge economic costs.

Given the ubiquitous nature of OC traits, they might play a key role in the etiopathogenesis of several OC-related mental disorders, possibly contributing to their onset and chronicity (Fontenelle et al., 2020; Snorrason et al., 2021). Indeed, OC traits might represent an important risk factor for the development of OC-related manifestations and disorders, especially when high-risk individuals encounter environmental stressors (Destrée et al., 2023). For instance, during COVID-19 pandemic a widespread and aspecific increase in OC behaviors has been observed, even in healthy individuals (Albertella et al., 2021; Dong et al., 2020; Elhai et al., 2020; Ornell et al., 2021; Tanir et al., 2020). Despite rigorous efforts for investigating cognitive and neurobiological processes underlying the onset and maintenance of OC traits have been made, evidence is still mixed and often inconclusive (for reviews, see Ferreira et al., 2020; Hezel and McNally, 2016).

From the cognitive viewpoint, individuals with OC traits were found to share alterations in functions falling under the cognitive control domain, broadly defined as the ability to coordinate information processing and actions complying with behavioral goals (Botvinick et al., 2001; Cohen, 2017). Specifically, cognitive control sub-functions thought to play a role in OC disorders include behavioral and cognitive inhibition, cognitive flexibility, conflict resolution and decision making. Indeed, the neuropsychological profile of patients with OC disorders was found to be predominantly characterized by impairments in cognitive flexibility, interference resolution, inhibitory control and decision-making (Ferreira et al., 2020; Hezel and McNally, 2016; Nisticò et al., 2021; Norman et al., 2019; Perera et al., 2023; Snyder et al., 2015; Shin et al., 2014). Specifically, moderate impairments of conflict resolution (i.e., ability of processing task-relevant information when perceiving a stimulus and/or selecting a response, simultaneously ignoring interfering task-irrelevant information and/or inhibiting prepotent responses; Nee et al., 2007, Tafuro et al., 2019), along with neurofunctional alterations of brain areas involved in conflict detection and resolution, have been consistently reported (see Ahmari and Rauch, 2022; Martínez-Esparza et al., 2021, for an overview). Notably, similar impairments have been observed both in healthy relatives of patients with OC disorder and in patients during the premorbid phase (Bora, 2020; Cavedini et al., 2010; Chamberlain et al., 2007). Additionally, cognitive control deficits have been also found in other compulsive-impulsive populations, like those affected by social network addiction (Müller et al., 2021), internet addiction (Ioannidis et al., 2019), internet gaming addiction (Yao et al., 2022; Zhou et al., 2012), binge eating (Córdova et al., 2017), and compulsive shopping (Lindheimer et al., 2020). Interestingly, cognitive control impairments are hypothesized to be trans-diagnostically impaired in several mental disorders (McTeague et al., 2016).

Therefore, deficits in cognitive control might not just be one of the features associated with OC traits. Rather, they might be at the *core* of several OC phenomena typically falling under other domains (e.g., subjective experience, mental representations and, eventually, behavior).

Concerning cognitive control, the dual mechanism of control (DMC) hypothesis (Braver, 2012) distinguishes between two forms of control: *proactive* and *reactive*. Specifically, proactive control is a tonic process underlying the ability to actively sustain task-relevant representations in working memory *before* the occurrence of conflicting information, biasing perception and action accordingly. This control mode is implemented in an anticipatory fashion to prioritize the task-relevant information over the task-irrelevant one. That is, by representing the abstract structure of the task (e.g., overall proportion of congruent stimuli), we can funnel in advance our cognitive resources and, eventually, adjust our behavior accordingly (i.e., I expect congruent stimuli to occur with a specific probability, therefore I would adjust cognitive resources and behavior according to that specific probability of encountering conflicting information). In contrast, reactive control is a phasic process underlying the ability to transiently retrieve and reactivate task-relevant representations only *after* conflict has been detected. That is, if specific conflict-eliciting events occur with different structured probabilities, each event-specific representation and, eventually, behavior would be affected accordingly. Nonetheless, if different probabilities are associated with specific events, the behavior can be adjusted only *after* stimulus has occurred. In other words, it is a late correction mechanism triggered by conflict detection. Evidence suggests that these two mechanisms work independently and simultaneously (Mäki-Marttunen et al., 2019) and are underlain by dissociable neurofunctional substrates (Braver et al., 2007; Burgess and Braver, 2010; Cohen and Cavanagh, 2011; Cavanagh and Frank, 2014; De Pisapia and Braver, 2006; West and Bailey, 2012, but see Viviani et al., 2023c).

Given the literature about typical OC phenomena (e.g., pathological doubt and uncertainty; Chiang and Purdon, 2023; Lunn et al., 2023; Pinciotti et al., 2021; Vazard, 2021; Williams and Levinson, 2021), some OC manifestations might arise from abnormalities in judging when, how and to what extent implementing control processes or actions for obtaining a specific outcome (i.e., proactive control). In other words, the pathological uncertainty observed in OCD might derive from deficits in accurately representing the structure of the environment and/or in using representations to implement adaptive behaviors accordingly. In turn, this might reflect an impairment in forming, adapting and/or maintaining relevant representations according to environmental demands. This might lead to questioning one’s own representations (reflected in pathological doubt and uncertainty), eventually hindering the proper implementation of goal-directed processes and behaviors. These hypotheses are supported by recent Bayesian models describing OC phenomena as abnormalities in representing, correcting and adaptively using action-outcome state transitions (see Fradkin et al., 2020a, 2020b; Sharp et al., 2021, for a detailed description). Nonetheless, these studies investigated hypotheses by means of in-silico simulations, and empirical confirmation is still lacking or controversial (e.g., Fruehauf et al., 2021).

Thus, the present study is aimed at investigating how OC traits are associated with alterations of *proactive* control when implemented for conflict resolution. Specifically, we hypothesized that OC traits might be associated with impairments in representing the probabilistic context reflecting the environmental requirements for proactive control. Notably, a deep understanding of how cognitive control impairments and OC phenomena are related to each other could be relevant for developing and testing innovative prevention and/or intervention strategies, eventually improving the quality of life of OC populations.

To this aim, we stratified our sample according to the severity of their OC traits. Afterwards, we administered the participants with the Stroop task (MacLeod, 1991; Stroop, 1935) to assess conflict resolution. Specifically, we used the perifoveal spatial Stroop task developed by Viviani et al. (2023a). Notably, the spatial Stroop tasks have been already used successfully to detect the neurofunctional substrates of proactive control (Tafuro et al., 2019; Visalli et al., 2023; Viviani et al., 2023a, 2023b). Importantly, the demands for proactive control were systematically manipulated by changing the probabilistic context (for further details, see Methods section). Consequently, we expected the Stroop effect to vary consistently with proactive control requirements. That is, in contexts characterized by higher control demands, more cognitive resources are recruited and, therefore, the Stroop effect should be less pronounced, compared to contexts characterized by lower control demands.

Additionally, we used a novel approach that allowed us to further manipulate proactive control on a trial-by-trial basis (see Viviani et al., 2023a, 2023b, 2023c). Interestingly, this approach was proven to be effective in overcoming design-level limitations in simultaneously manipulating different variables and in minimizing the effects of low-level confounds (see Viviani et al., 2023a, 2023c for a detailed discussion).

Finally, concerning specific hypotheses, we expect this LWPC-dependent modulation of the Stroop effect to be reduced in participants with more severe OC traits, given the hypothesized impairment in proactive control.

## Materials and methods

### Participants

Participants were recruited among psychology students at the University of Padova (Padova, Italy), through advertisements on social media and through snowball sampling. Both Italian and English-speaking participants were included. A total of 242 participants completed the screening phase. During this phase, participants were administered online versions of the Obsessive Compulsive Inventory (OCI) and the Depression-Anxiety-Stress Scale (DASS-21, detailed below) to measure the severity of obsessive compulsive traits and possible comorbid symptoms (i.e., depression, anxiety, stress). Participants were required to subscribe the informed consent to participate in the study, which fulfills the ethics standards of the 2013 Declaration of Helsinki for human studies. Subsequently, we selected participants according to their OCI scores, following a stratified sampling procedure. Specifically, we divided the OCI scores into equal-width intervals and we selected participants in order to obtain homogeneous subsamples—in terms of size and variance—for each layer while sampling the full continuum of compulsive traits.

After screening, a total of 104 participants (70 females, 33 males, 1 other; mean age = 26.1 years, SD = 6.6; 83 right-handed, 73 Italian native speakers) met inclusion criteria (i.e., no current or past history of psychiatric or neurological disorders, no recent assumption of alcohol or drugs possibly affecting cognitive functions) and were selected to perform the task. Descriptive statistics about the distribution of OCI and DASS-21 scores are reported in Table 1. At the moment of data collection, the project had been already approved by the Ethical Committee for the Psychological Research of the University of Padova (approved protocol reference number: 4631) and completed a behavioral task online between April 2022 and October 2022.

**Table 1:** Descriptive statistics for the Obsessive-Compulsive Inventory (OCI) and the Depression-Anxiety-Stress Scale (DASS-21) scores.

		*M*	SD
OCI	Washing	0.75	0.63
	Checking	0.74	0.59
	Ordering	1.09	0.99
	Obsessions	0.94	0.86
	Hoarding	0.86	0.70
	Mental neutralizing	0.86	0.82
	Tot	0.61	0.55
DASS-21	Depression	11.79	9.93
	Anxiety	6.73	6.45
	Stress	11.79	9.93
	Tot	33.40	22.60

M, mean; SD, standard deviation, Tot, total scores.

To determine the minimum sample size, we performed an *a priori* power analysis in G*Power (Erdfelder et al., 1996) for the interaction of main theoretical interest regarding the LWPC-dependent proactive control modulation of the Stroop effect, with a 2 (Congruency: Congruent vs. Incongruent) × 3 (block-level LWPC: 30, 50, and 70%) repeated measure ANOVA, assuming an alpha of 0.05, a power of 0.80 and correlation between repeated measures of 0.75. The power analysis revealed that a sample size of 82 was enough to detect a significant interaction with a SESOI of Cohen’s *d* = 0.2. We also performed a similar power analysis using GLIMMPSE (Kreidler et al., 2013), which allowed us to compute the statistical power for a linear mixed-effect (LMM) model more similar to that we used in our main analysis, that is, one with the same Congruency and LWPC factors, but also including a categorical covariate (low- vs. high-OCI) to model the OCI modulation of the Congruency by LWPC interaction; This analysis also revealed a required sample size of 82. It should also be noted that LMMs tend to provide higher power than other more standard statistical approaches (de Melo et al., 2022; Yang et al., 2014).

Another *a priori* power analysis was conducted for the OCI modulation of the participants’ proactive control and interference resolution abilities, which is assessed by the correlation between participants’ OCI scores and their Congruency and LWPC by Congruency effects estimated by the LMM, with a smallest effect size of interest (SESOI) of *r* = 0.31623, corresponding to 10% of explained variance, assuming an alpha of 0.05 and a power of 0.80. This analysis revealed that 58 participants were needed. Finally, an *a priori* power analysis for the equivalence test (Two One-Sided Test, TOST; Lakens, 2017; Lakens et al., 2020; Meyners, 2012; testing for the practical absence of a significant correlation between participants’ OCI and their proactive control ability, see below), with the same assumed alpha and power levels, revealed a minimum sample size of 83 participants.

Of note, we decided to recruit a larger sample than that indicated by these power analyses, so as to be able to estimate the effects of interest with greater precision and detect them with greater statistical power. Indeed, our final sample size of 104 participants allowed us to find a significant LWPC by Congruency effect, a significant correlation between participants’ OCI scores and their cognitive control abilities, and a significant TOST for this correlation, with the above-mentioned smallest effect sizes of interest, with a power >0.89, 0.95, and 0.90, respectively.

### Materials and procedure

#### Obsessive compulsive inventory (OCI)

The *Obsessive Compulsive Inventory* (OCI, Foa et al., 1998) is a multidimensional self-report questionnaire developed to quantify obsessive-compulsive symptoms. It consists of 42 items divided into seven subscales, specifically: (1) *Washing* (eight items, e.g., “I think contact with bodily secretions [perspiration, saliva, blood, urine, etc.] may contaminate my clothes or somehow harm me.”); (2) *Checking* (nine items, e.g., “I repeatedly check doors, windows, drawers etc.”), (3) *Doubting* (three items, e.g., “I ask people to repeat things to me several times, even though I understood them the first time.”), (4) *Ordering* (five items, e.g., “I need things to be arranged in a particular order.”), (5) *Obsessing* (eight items, e.g., “I have thoughts that I might want to harm myself or others.”), (6) *Hoarding* (three items, “I collect things I do not need.”), (7) *Mental neutralizing* (six items, “I need to pray to cancel bad thoughts or feelings.”). Each item is rated according to a 5-point Likert scale (0 = not at all; 4 = extremely). The participant is asked to answer according to the distress experienced during the past month with reference to the described phenomenon. The OCI scale and its subscales have overall good reliability (Cronbach’s *α*; OCI tot: 0.86–0.95, OCI scales: >0.70), good test–retest reliability (0.68 > *r*_t-t_ > 0.90) and good convergent and divergent validity (Foa et al., 1998). The questionnaire was administered in English (Foa et al., 1998) to non-Italian native speakers and in Italian (Sica et al., 2009) to Italian native speakers.

#### Depression-anxiety-stress scale (DASS-21)

The *Depression-Anxiety-Stress Scale* (DASS-21; Antony et al., 1998; Henry and Crawford, 2005) is a short 21-item form of the original DASS scale (Lovibond and Lovibond, 1995). It is designed at measuring the severity of anxious-depressive and stress-related symptoms, either in clinical or in nonclinical samples. Specifically, we administered the DASS-21 to control for confounding effects of depression, anxiety and/or stress on possible OCI effects. The DASS-21 includes three subscales (seven item each), specifically: (1) *Depression* (dysphoria, hopelessness, devaluation of life, self-deprecation, lack of interest/involvement, anhedonia and inertia, e.g., “I felt that I had nothing to look forward to”); (2) *Anxiety* (autonomic arousal, skeletal muscle effects, situational anxiety, and subjective experience of anxious affect; e.g., “I felt I was close to panic”) and (3) *Stress* (chronic non-specific arousal; e.g., “I found myself getting agitated”). The participant is required to rate how much the statement applies to himself/herself referring to the past week. The answer is given through a 4-point Likert scale (0 = did not apply to me at all; 3 = applied to me very much or most of the time). Both total and partial scores have good internal consistency (Cronbach’s *α*: 0.88 for the Depression scale; 0.82 for the Anxiety scale; 0.90 for the Stress scale, and 0.93 for the total score), and good factorial solution (eigenvalues 9.07, 2.89, and 1.23, respectively, accounting for 67% of the variance). The questionnaire was administered in English and in Italian (Bottesi et al., 2015) to Italian native speakers.

#### Perifoveal spatial Stroop task

After screening, the selected participants were administered online with an adapted version of the perifoveal spatial Stroop task (Viviani et al., 2023a). The probabilistic context was manipulated as illustrated below. The experiment was programmed using Psytoolkit (Stoet, 2010, 2017). Participants were given a link and were asked to complete the task on their own, in a quiet and silent environment.

The task was executed in full-screen mode. Stimuli were presented on a 1,024 × 768 pixels screen colored with a light sky-blue background (RGB: 189, 215, 238). At the beginning of each trial, a fixation stimulus—consisting of a partial black square outline (94 × 94 pixels) enclosing a black fixation cross (30 × 30 pixels)—was presented. Previously, participants had been instructed to fixate the cross and to keep their gaze at that screen position. After 500 ms, a target stimulus consisting of a thick arrow pointing to one of four possible directions (upper-left, upper-right, lower-left or lower-right) appeared in one of the four positions outlined by the elements characterizing the previously-described fixation stimulus (i.e., upper-left, upper-right, lower-left, or lower-right corner). Notably, the arrow direction could be consistent (i.e., congruent) or inconsistent (i.e., incongruent) with the position (i.e., the corner) at which it appeared. Participants were instructed to respond according to the *direction* pointed by the arrow, independently from its position on the screen. Therefore, the task required the participant to focus on the direction (task-relevant feature), while ignoring the position (task-irrelevant feature) of the stimulus to respond correctly. The target lasted until the participant’s response, up to a maximum of 2,000 ms. Participants were instructed to provide their response by pressing one of four keys (D, C, K or M) with their fingers (left middle, left index, right middle or right index finger, respectively) on their computer keyboard. Specifically, the spatial arrangement of the keys reflected the spatial arrangement of both task-relevant (direction) and task-irrelevant (position) features of the stimuli. This choice was made to ensure a complete Stroop effect, including response conflict, by establishing a dimensional overlap between all the task dimensions (i.e., relevant/irrelevant stimulus features and response dimensions), eventually creating a type-eight ensemble (Kornblum, 1992; Viviani et al., 2023b). Participants were instructed to position their fingers on the corresponding buttons before starting each block and to hold them in place until the end of the block. Thereafter, a blank screen with a light sky-blue background was presented for 500 ms. Subsequently, a new trial started.

**Figure 1:**
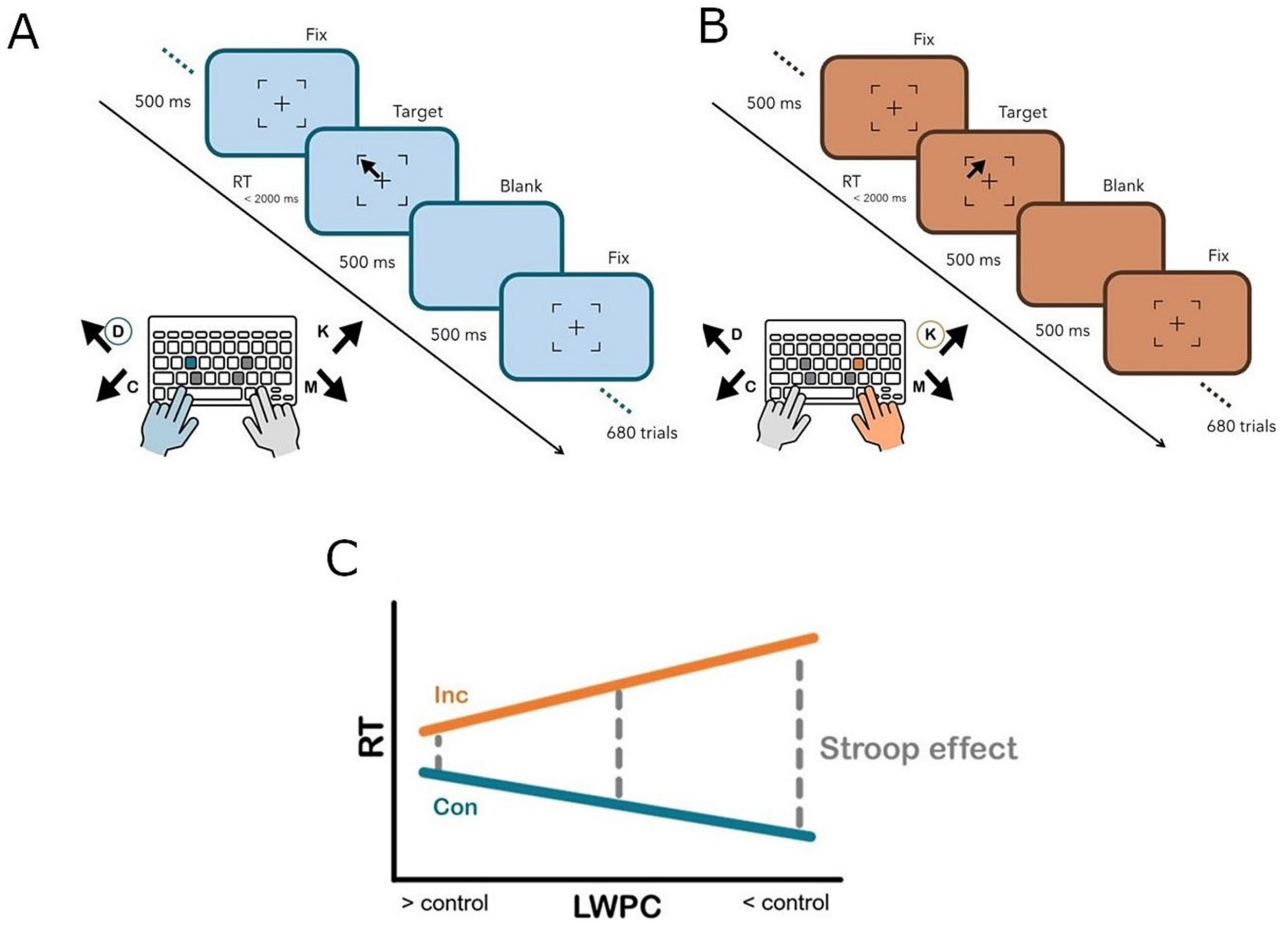
Experimental paradigm illustrating a congruent **(A)** and incongruent **(B)** trial, respectively. Participants were asked to answer according to the direction of the arrow and to ignore its position within the square surrounding the fixation cross. The response was given by pressing one of four keys (D, C, K, M) on a canonical computer keyboard, which were spatially arranged to overlap with the four stimulus directions and positions. Specifically, participants were asked to respond with the index (C, M) or middle (D, K) finger of the left (D, C) or right (K, M) hand according to the arrow direction. **(C)** Experimental manipulation. When the proportion of congruent trials is lower (low LWPC), the elicited control is higher and the Stroop effect (difference in terms of response times between congruent and incongruent trials, gray dashed line) is less pronounced. *Vice-versa*, when the proportion of congruent trials is higher (high LWPC), the elicited control is lower and the Stroop effect is more pronounced. Con, Congruent (blue solid line); Inc, Incongruent (orange solid line); LWPC, list-wide proportion congruency; RT, response times.

Intrinsic requirements for proactive control (Gonthier et al., 2016) were operationalized and manipulated by systematically varying the proportion of congruent trials in a list-wide fashion (list-wide proportion congruency; LWPC; see Figure 1). In other words, we changed the probabilistic context, eventually creating different levels of proactive control demands (Bugg and Hutchison, 2013; Gonthier et al., 2016; Visalli et al., 2023). Indeed, when a block is characterized by a higher proportion of congruent trials (high LWPC), conflict occurs less frequently, therefore the demand for implementing proactive control is lower. In contrast, when the proportion of congruent trials is lower (low LWPC), conflict occurs (and must be solved) more frequently. In other words, control demands are higher, thus more cognitive resources are typically funneled for implementing proactive control (Bugg and Hutchison, 2013; Gonthier et al., 2016; Visalli et al., 2023).

Consequently, the Stroop effect is expected to be reduced in contexts where LWPC is lower (higher control demands) compared to contexts in which the latter is higher (lower control demands). Specifically, we assumed that in blocks characterized by a higher probability of incurring in incongruent trials (low LWPC), cognitive control implemented in an *anticipatory* fashion (i.e., proactive control) should be facilitated. On the other hand, when the list-wide probability of incurring in incongruent trials is low (high LWPC), it would be inconvenient for the system to implement proactive control, as the latter is a really onerous process in terms of cognitive resources. Therefore, the implementation of proactive control in this case would be attenuated or hindered. This approach was proven to be more effective for manipulating requirements proactive control than traditional ones (e.g., see Braem et al., 2019; Visalli et al., 2023; Viviani et al., 2023c).

**Figure 2:**
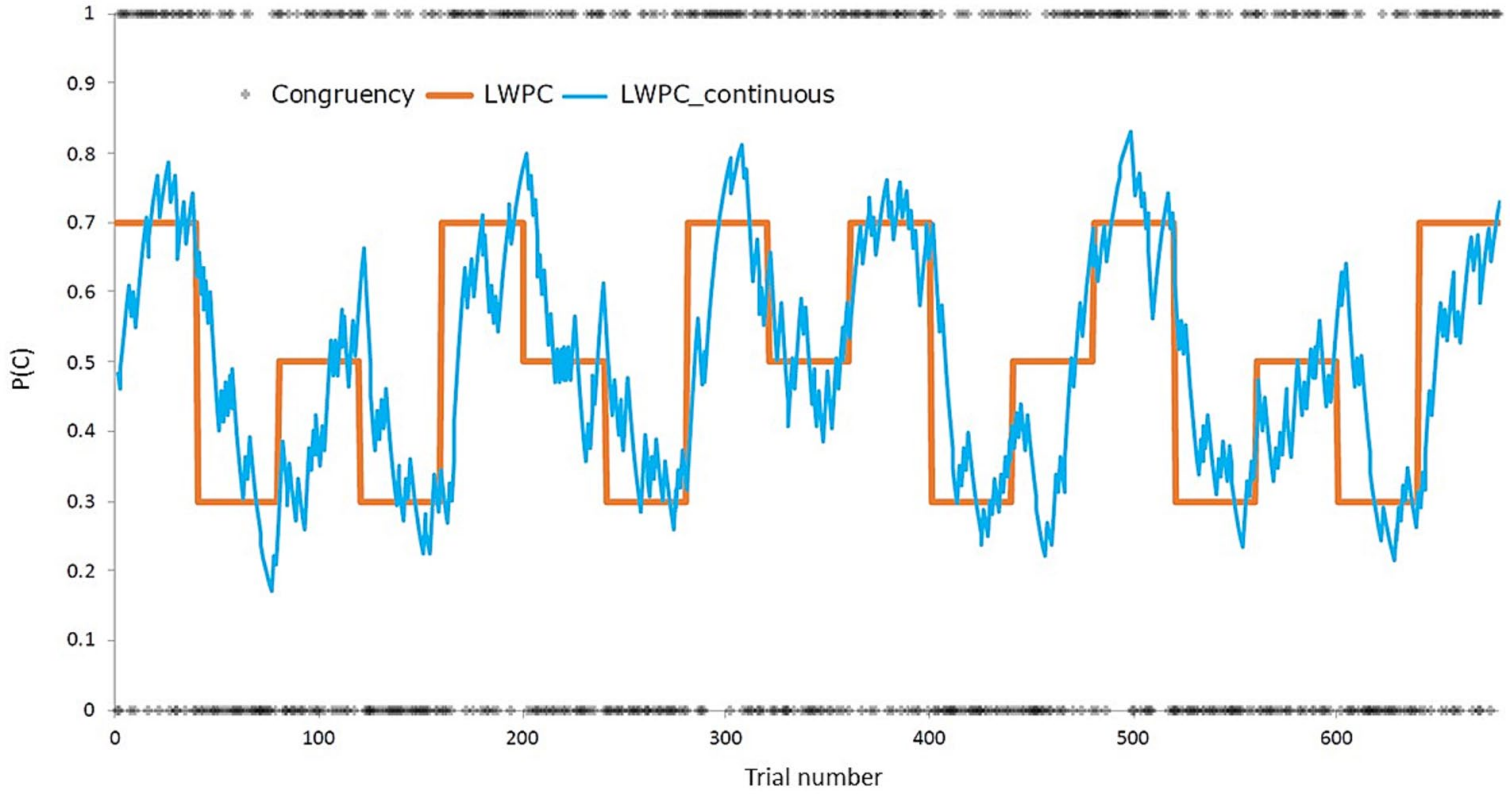
Depiction of the experimental manipulation of the list-wide proportion of congruency (LWPC), both before (orange solid line, block-wise) and after (blue line, trial-wise) applying the Hierarchical Gaussian Filter (Mathys et al., 2011). P(C), proportion of congruency.

Concerning the trial-list design, the task was divided into five or six blocks per condition (block-level LWPC: 70, 50, 30%), for a total of 680 trials (17 40-trial blocks). List-wide probabilities were presented in a balanced order. The order of trials was pseudorandomized within each block using the Mix software (van Casteren and Davis, 2006) for avoiding first-order priming effects. A total of 1,000 trial-lists were generated. Then, trial-level LWPC and probability of response given a stimulus (PRS) were computed for each trial-list by applying the Hierarchical Gaussian Filter (for a detailed description, see Mathys et al., 2011; Visalli et al., 2023). In short, the HGF allows to update the expected probability of the occurrence of an event, that is, the probability of target congruency for trial-level LPWC (hereafter, continuous LWPC, cLWPC) according to the past history of their specific occurrences, in a trial-wise fashion. Notably, since participants were blind to the probabilistic structure of the task, we assumed that the *estimated* probability of congruency (PC) evolved *within* the block and substantially differed between the initial and the final trials of the same block, regardless of its discrete PC value. Therefore, the HGF allowed us to create a *continuous PC*, an index representing the trial-wise probability of target congruency. A graphical representation of the HGF estimation is shown in Figure 2.

Moreover, we used the HGF to compute trial-wise probabilities of PRS and of the stimulus location and the response, which were included as predictors of non-interest in the statistical model (see Statistical analyses). Specifically, we computed PRS to account for the effect of contingency on performance, as previous studies demonstrated that PRS partially overlaps with the effect of interest, eventually masking it (see” contingency hypothesis,” Schmidt, 2019; Schmidt and Besner, 2008). To this end, the trial-list with the lowest correlation between trial-level LWPC and PRS was used for all the participants. In the selected trial-list, the collinearity between trial-level LWPC and PRS variables was *r* = 0.12 (corresponding to 1.5% of shared variance).

Precise and simple instructions were provided before the beginning of the task. After instructions were given, a training block started (block-level LWPC = 50%) and participants received feedback about the correctness and the speed of their responses. Practice trials stopped when the participant reached an accuracy of 75% within 16 trials (after at least six trials). The task included 680 trials, interleaved by a 60-s break after every 170 trials, for a total of four breaks.

### Statistical analyses

Inverse-transformed response times (iRTs: −1,000/RT) were analyzed by means of linear mixed-effect models (LMMs) in R^1^ using the lme4 library (Bates et al., 2015). Specifically, we computed *inverted* RTs to account for the positive skewness of the RTs distribution (Ambrosini et al., 2019; Brysbaert and Stevens, 2018; Viviani et al., 2023a). As common practice in the literature, RTs in the first trial at the beginning of each block, as well as the RTs in error trials (i.e., incorrect key pressing according to the presented target) and in post-error trials, as well as trials with missed responses, were excluded from the analyses (10% of excluded trials).

Concerning the LMMs, initially we detailed a full LMM accounting for all experimental effects and possible low-level confounds known to affect trial-wise iRTs variability (Baayen and Milin, 2010; Visalli et al., 2023).

Specifically, we included in the fixed part: (1) congruency (two-level factor, congruent and incongruent), (2) the cLWPC and (3) the OCI z-scores (zOCI) as continuous predictors, as well as their three-level interaction (Congruency × cLWPC × OCI z-scores and related low-order interactions). Moreover, as low-level confounding variables the fixed part included: (4) the rank-order of each trial (Trial) and (5) the iRT of the preceding trial (pre_iRT) to account for time-dependent (e.g., learning and fatigue) effects; (6) the horizontal and vertical coding of the arrow direction (hDIR and vDIR, respectively) to account for potential effects related to the response hand and/or finger, respectively; (7) the horizontal and vertical coding of the arrow location (hPOS and vPOS, respectively) to account for potential effects related to left/right and/or upper/lower visual field differences, respectively; finally (8) trial-level contingency (cPRS), (9) probability of the stimulus location (cPL), and (10) probability of the response (cPR) were included to account for potential effects of perceptual and/or motor confounding probabilistic contexts.

The random part included: participant-specific correlated random intercepts and slopes for the Congruency × cLWPC interaction and related low-order interactions and main effects. Therefore, the final model was specified as the following Wilkinson-notation formula (Equation 1):


(1) $$\begin{array}{l} iRT\sim \mathrm{Trial}+pre\;iRT+\mathrm{cPRS}+cPL+cPR+\mathrm{hPOS}+\mathrm{vPOS}\\ \;+\mathrm{hDIR}+\mathrm{vDIR}+\mathrm{Congruency}\times \mathrm{cLWPC}\times \mathrm{zOCI}\\ \;+\left(\mathrm{Congruency}\times \mathrm{cLWPC}\;|\;\mathrm{Participant}\right) \end{array}$$

After fitting the LMM model, we inspected its residuals to assess whether there was evidence of stress in the model fit and, in that case, the model was refitted after excluding trials characterized by absolute standardized residuals exceeding 2.5 SD (Baayen and Milin, 2010).

Finally, in case of null results, we tested for the absence of a significant OCI modulation of participants’ cognitive control abilities (i.e., the correlation between participants’ OCI scores and both their general interference resolution abilities and their LWPC-dependent proactive control abilities, as assessed, respectively, by their random slopes for the Congruency and the LWPC by Congruency effect, both estimated by the LMM). To this aim, we performed TOST equivalence tests (Lakens, 2017; Lakens et al., 2020; Meyners, 2012). Specifically, the TOST compares the observed effect with the smallest effect of interest (SESOI) examining whether the former falls within a region around 0 delimited by a upper and a lower bound—defined according to the SESOI—describing two extreme regions containing 90% of observations if alpha is set to 0.05. If the observed effect falls in the area around 0, the alternative hypothesis (in this case, the *absence* of effect) is confirmed. The TOST was performed using the Excel spreadsheet developed by Lakens (2017; available at the following link: https://daniellakens.blogspot.com/2016/12/tost-equivalence-testing-r-package.html).

## Results

The accuracy was high (*M* = 0.94; SD = 0.07), especially for congruent (*M* = 0.98; SD = 0.05) but also for incongruent (*M* = 0.90; SD = 0.10) trials. Inverted response times (iRTs) were consistent with the literature about interference resolution tasks (overall: *M* = −1.96, SD = 0.30; congruent: *M* = −2.89, SD = 0.32; incongruent: *M* = −1.72, SD = 0.32). Moreover, differences between congruent and incongruent trials (i.e., Stroop effect)—both in accuracy (*M* = 0.78, SD = 0.09, t = 9.17; *p* < 0.0001) and in iRTs (*M* = 0.47, SD = 0.11, *t* = 42.48; *p* < 0.0001)—were statistically significant. For our analyses, we focused on iRTs (see also Visalli et al., 2023).

### Linear mixed-effect model (LMM)

As described in the method section, we report the results of the trimmed LMM model that was refitted after excluding observations with absolute standardized residuals exceeding 2.5 SD (2.06% of trials), which achieved a sufficient closeness to normality of residuals. For a summary of LMM results, see Table 2.

**Table 2:** Parameters of the main effects and interaction for the predictors of specified model (see Equation 1).

Predictors	Estimates	CI	*p*
(Intercept)	−1.92	−2.00 to −1.85	**<0.001**
Trial	−0.13	−0.14 to −0.13	**<0.001**
PRS	−0.05	−0.06 to −0.05	**<0.001**
PR	−0.11	−0.12 to −0.10	**<0.001**
PL	-0.1	−0.14 to −0.07	**<0.001**
hPOS	−0.01	−0.02 to −0.01	**<0.001**
vPOS	0.01	0.00 to 0.02	**0.002**
hDIR	−0.05	−0.05 to −0.04	**<0.001**
vDIR	−0.09	−0.09 to −0.08	**<0.001**
pre iRT	0.08	0.08 to 0.09	**<0.001**
Congruency	−0.42	−0.44 to −0.39	**<0.001**
cLWPC	0.06	0.05 to 0.07	**<0.001**
zOCI	−0.02	−0.09 to 0.04	0.443
Congruency × cLWPC	−0.1	−0.12 to −0.09	**<0.001**
Congruency × zOCI	0.02	−0.01 to 0.04	0.162
cLWPC × zOCI	0	−0.01 to 0.01	0.899
Congruency × cLWPC × zOCI	0	−0.01 to 0.01	0.591
Marginal *R*^2^/Conditional *R*^2^	0.303/0.647		

CI, confidence interval; cLWPC, continuous list-wide proportion congruency; hDIR, horizontal direction; hPOS, horizontal position; PL, probability location; PR, probability response; PRS, probability response|stimulus; pre iRT, preceding inverted response time; vDIR, vertical direction; vPOS, vertical position; Trial, trial number; zOCI, z-converted Obsessive-Compulsive Inventory score. Bold values denote statistical significance (p < .05).|stimulus; pre iRT, preceding inverted response time; vDIR, vertical direction; vPOS, vertical position; Trial, trial number; zOCI, z-converted Obsessive-Compulsive Inventory score. Bold values denote statistical significance (*p* < .05).

**Figure 3:**
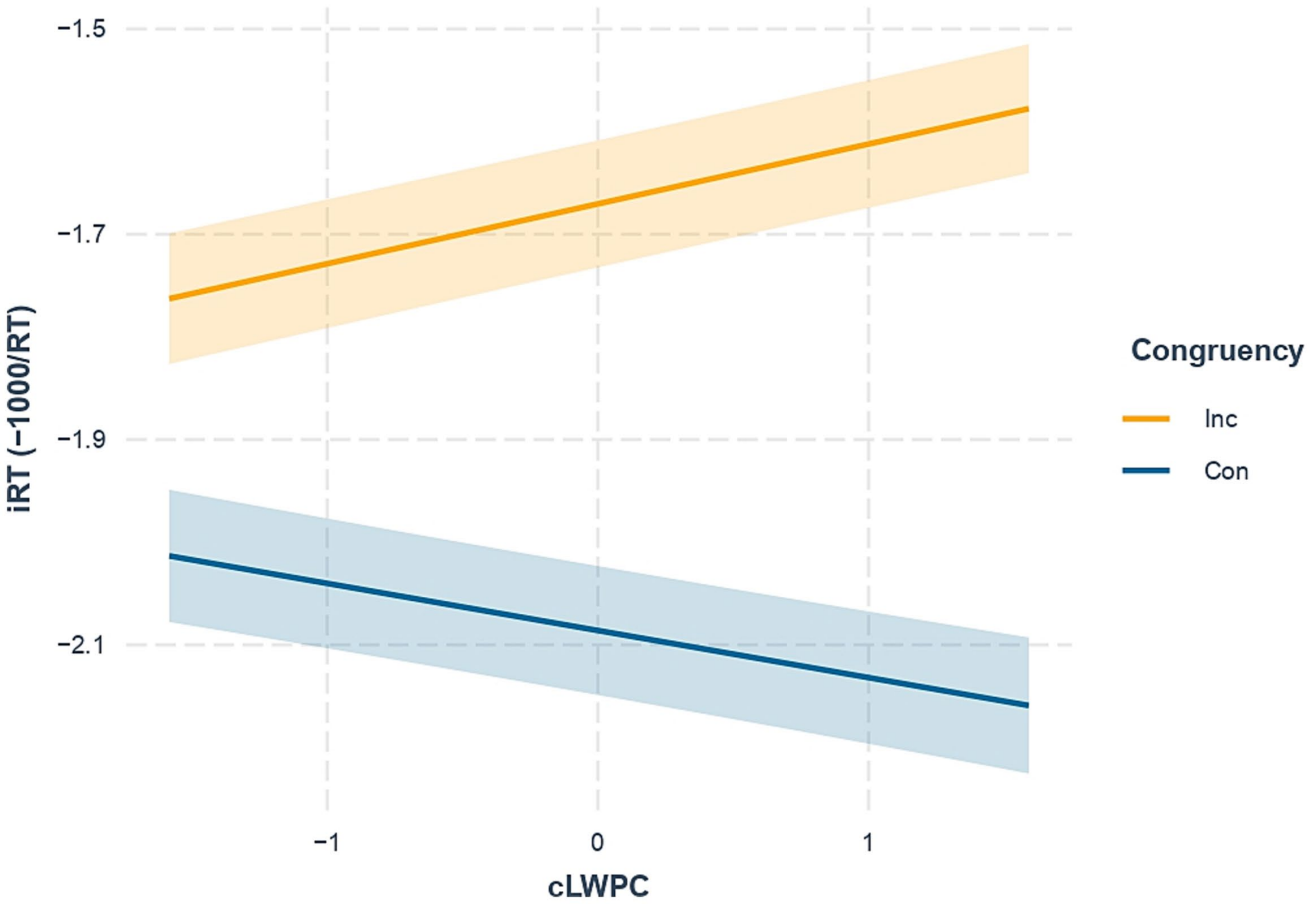
Effect of the interaction between congruency and trial-wise list-wide proportion congruency (Congruency × cLWPC). Con, Congruent trials (blue solid line); Inc, Incongruent trials (orange solid line). iRT, inverted response times. Shaded error bars indicate standard errors of estimated marginal means.

The hypothesized two-way interaction between congruency and proactive control (Congruency × cLWPC) was significant. In other words, environmental requirements for proactive control modulated the Stroop effect in the expected direction (i.e., less marked difference in iRTs between congruent and incongruent trials in case of low cLWPC and vice versa for high cLWPC, see Figure 3).

Nonetheless, the effect of total OCI scores, as well as their two- and three-way interactions with the experimentally-manipulated variables, were not significant.

### Equivalence test (TOST)

The TOST resulted to be statistically significant for the correlations between participants’ OCI scores and both their general interference resolution abilities, as assessed by their random slopes for the Congruency effect estimated by the LMM (*r* = 0.137, CI_low_ = −0.025, CI_high_ = 0.293, *p* = 0.042) and their LWPC-dependent proactive control abilities, as assessed by their random slopes for the LWPC by Congruency effect estimated by the LMM (*r* = 0.093, CI_low_ = −0.070, CI_high_ = 0.252, *p* = 0.015). In other words, this analysis revealed that the observed correlations between participants’ OCI scores and both their Congruency and their LWPC by Congruency effects were statistically equivalent to 0. Similar TOST analyses were performed on the Spearman’s correlation coefficients, to control for potential violations of the statistical assumptions required for parametric tests, confirming the results observed for the Pearson’s correlations (Congruency: *r* = 0.089, CI_low_ = −0.075, CI_high_ = 0.247, *p* = 0.013; LWPC by Congruency: *r* = 0.059, CI_low_ = −0.207, CI_high_ = 0.218, *p* = 0.006). Finally, since the effect of OCI on performance was statistically equivalent to zero, we did not control for possible confounding effects of DASS-21 factors.

## Discussion

Our study was aimed at investigating if and how subclinical OC traits were related to specific alterations in proactive control. To this end, we sampled the full continuum of OC traits in the general population and assessed their ability to solve conflict in a proactive fashion. Specifically, the administered Stroop task implicitly induced the participant to represent the probabilistic structure of the environment—in terms of congruency—to solve conflict and respond optimally. Given the cognitive and behavioral phenomena associated with OC traits, we expected individuals with more severe (although sub-clinical) OC traits to show alterations in forming or maintaining and, eventually, in adaptively using those task-relevant representations to solve conflict. Therefore, we hypothesized that an increased severity of OC traits could have been associated with a reduced modulation of performance according to probabilistically-manipulated requirements for proactive control (LWPC). In operational terms, this could be translated into a less marked difference in terms of Stroop effects between low and high LWPC trials.

Contrary to our hypothesis, we found that OC traits were not significantly associated with behavioral performance. Then, we tested for the absence of such effects and, indeed, we found that they were significantly equivalent to zero. Therefore, we can conclude that OC traits are not associated with the implementation of proactive control to solve conflict, at least not in the context of the Stroop task used here on a non-clinical sample.

Our results suggest that, contrary to the literature positioning cognitive control abnormalities at the core of compulsive disorders (Nisticò et al., 2021; Norman et al., 2019; Perera et al., 2023; Snyder et al., 2015; Shin et al., 2014), OC phenomena—at least at the subclinical level—are not related to abnormalities in proactive control processes implemented for solving conflict. Specifically, clinical and experimental evidence suggests that OC manifestations might emerge from executive dysfunctions (i.e., conflict monitoring, attention, response inhibition); this hypothesis is further supported by neuroimaging evidence about abnormalities in frontal and fronto-striatal circuits underlying executive processes for OC populations compared to healthy controls (see Nisticò et al., 2021; Norman et al., 2019; Perera et al., 2023; Snyder et al., 2015; Shin et al., 2014 for reviews). Such inconsistencies between our findings and previous literature may have several explanations, whose nature might concern either the real underlying mechanisms of compulsive disorders or our methodological choices.

Concerning the former possibility (i.e., underlying mechanisms of compulsive disorders), from our results we must conclude that impairments in the implementation of proactive control for conflict resolution do not underlie subclinical OC traits. This is in line with studies which did not find any deficits in conflict resolution and cognitive control functions for compulsive populations, although evidence is mixed, and that is the reason why we wanted to test this relationship (for reviews, see Ferreira et al., 2020; Hezel and McNally, 2016). Importantly, assuming that OC traits are not underlain by proactive control deficits has important implications for developing new preventive strategies. For instance, clinical and experimental research should focus on potentiating other cognitive functions in subclinical populations, which might possibly play a more prominent role in the onset and maintenance of the OC-related disorder.

Moreover, we cannot exclude that impairments in other control processes or modes might underpin the development of compulsive disorders. For instance, *reactive* rather than proactive control processes might be impaired in OC populations. That is, OC manifestations might arise from abnormalities in properly implementing reactive control to solve conflict. Indeed, this might be in line with some clinical observations: for instance, individuals with OC traits may detect conflict or “disturbing” information in contexts where it is not even there (e.g., sensory phenomena like not-just-right experiences and incompleteness; Coles and Ravid, 2016; Ferrão et al., 2012; Horncastle et al., 2022; Melli et al., 2020). Therefore, given the outlined clinical phenomena, it would be worth investigating the relationship between OC traits and possible impairments in reactive control mechanisms in future research.

Furthermore, we might hypothesize that other processes falling into the cognitive control domain could be at the basis of OC traits and disorders. For instance, evidence about a bias and overreliance toward habitual at the expenses of goal-directed behavior has been extensively found (Banca et al., 2015; Gillan et al., 2016; Gillan and Robbins, 2014; Robbins et al., 2012; Voon et al., 2015). Indeed, habits share with compulsions the rigidity of the underlying action-outcome associations and the automaticity characterizing their learning and implementation. Therefore, an imbalance between the two processes might lead to issues in encoding the task structure (Seow et al., 2021) and, eventually, to massively implement stereotyped and repetitive actions. Nonetheless, experimental paradigms developed to investigate model-based and model-free control (e.g., sequential decision-making tasks) often make use of rewards, posing problems in dissociating ‘hot’ (reward-based) from ‘cold’ decision-making (Dayan and Berridge, 2014; Voon et al., 2017).

Nevertheless, given the diverse clinical pictures and phenomena characterizing compulsive disorders, alterations in several cognitive control mechanisms and even in specific sub-processes (e.g., detecting versus solving conflict) could be responsible for their onset and maintenance. Future studies should focus on thoroughly investigating cognitive control processes in populations with OC traits, possibly administering a complete battery of cognitive control tasks and trying to spot the behavioral endophenotype(s) associated with OC traits in a transdiagnostic fashion.

Regarding the methodological choices which might have affected our results, we must pinpoint several aspects eventually limiting their generalizability.

Firstly, participants recruited in the present study post-screening were not clinically diagnosed with an OC-related disorder. On one hand, this choice is preferable to control for possible clinical-related confounding variables (e.g., effects of medication, years from diagnosis, age of onset, etc.) and to investigate possible prodromic factors predisposing to the development of different OC-related disorders. On the other hand, our approach prevented us from investigating cognitive control abnormalities and/or clinical phenomena that need to exceed a certain threshold in terms of severity to be experimentally observable. Moreover, the sampling procedure adopted here might prevent the inclusion of specific categories of individuals, therefore reducing the generalizability of our results.

Secondly, the experimental procedure (both screening and experimental phases) were performed online and autonomously by participants. This procedure allowed us to reach a broader and somehow heterogeneous population, as well as to control for the experimenter’s effect. Nonetheless, this experimental approach severely limited our control over experimental conditions and possible confounds.

Thirdly, the investigation of the association of interest was limited to a very specific cognitive control process (i.e., conflict resolution) implemented in a very peculiar mode (i.e., forming, maintaining and proactively using behaviorally-relevant representations of the probabilistic structure of the environment). Although this methodological approach is optimal to increase task specificity for selectively assessing the process(es) of interest, it drastically reduces task sensitivity. In other words, other processes falling into the cognitive control domain (e.g., decision-making) and/or other control modes (e.g., reactive control) might be involved in the development and maintenance of compulsive disorders. Albeit our experimental procedure was not suited to investigate these working hypotheses, our work might inspire future studies conceived to more specifically test them.

Lastly, specific moral emotions (e.g., fear of guilt) are known to play a pivotal role in OC-related manifestations (Mancini and Gangemi, 2004). Indeed, the presence of stimuli eliciting obsessive concerns might heavily impact on cognitive processes and, importantly, on the ability to solve conflict (Mancini and Gangemi, 2004, 2018). Therefore, the choice to elicit conflict by means of emotionally neutral stimuli prevented us from investigating the potential influence of such secondary moral emotions on the ability to solve conflict. In other words, we cannot exclude that deficits in conflict resolution in OC populations may only arise following the exposure to stimuli evoking moral emotions related to the specific obsessive concern. Importantly, this might explain our findings about the absence of effects. Therefore, future studies should also address the influence of specific moral emotions, which might represent a precondition to observe abnormalities in conflict resolution for OC individuals.

## Conclusion

We found that OC traits were not associated with alterations of proactive control for conflict resolution. Several scientific explanations—also derived from specific methodological choices—might be at the basis of these results. Future research should address these issues by developing tailored methodologies accordingly. Firstly, assuming the presence of a to-be-reached severity threshold to observe an effect, a similar experimental procedure could be performed with clinical populations, controlling for the well-known clinically-relevant confounding variables. Additionally, the task should be adapted to control for possible ceiling effects and to increase the interindividual variability in cognitive performance. Lastly, other cognitive control processes and/or modes should be investigated and addressed, as OC traits have been consistently found, in the literature, to be associated with several executive deficits transcending the investigated probabilistic processes underlying the implementation of proactive control.

## Data Availability

The raw data supporting the conclusions of this article will be made available by the authors, without undue reservation.

## Footnotes

^1^ http://www.R-project.org/
